# Supplementary figures and images for: Organelle bottlenecks facilitate evolvability by traversing heteroplasmic fitness valleys
Source: Front Genet. 2022 Oct 28;13:974472. doi: 10.3389/fgene.2022.974472 (PMC9650085; doi:10.3389/fgene.2022.974472)

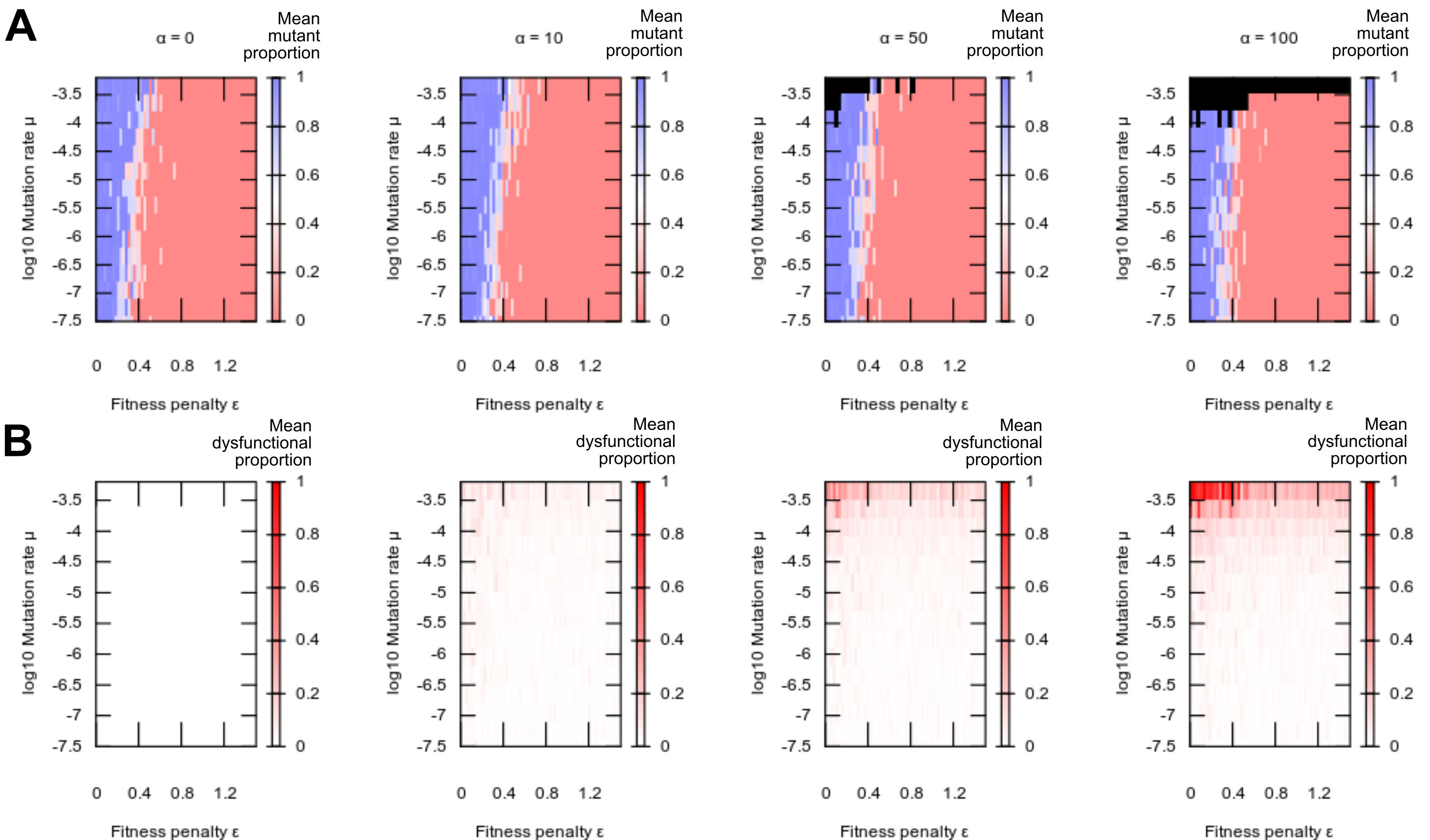

Supplement: Supplementary file 1 [file Image3.JPEG]

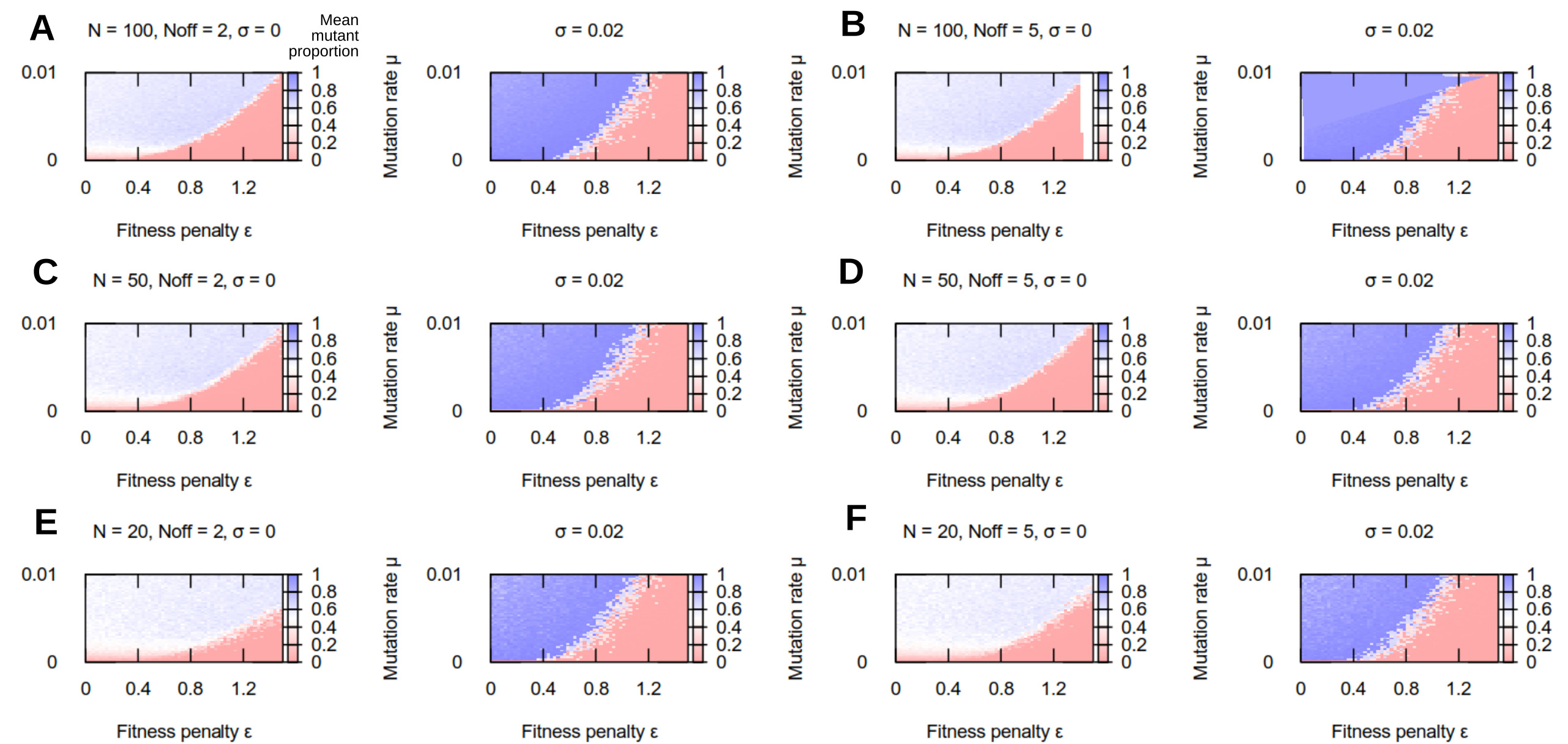

Supplement: Supplementary file 2 [file Image1.JPEG]

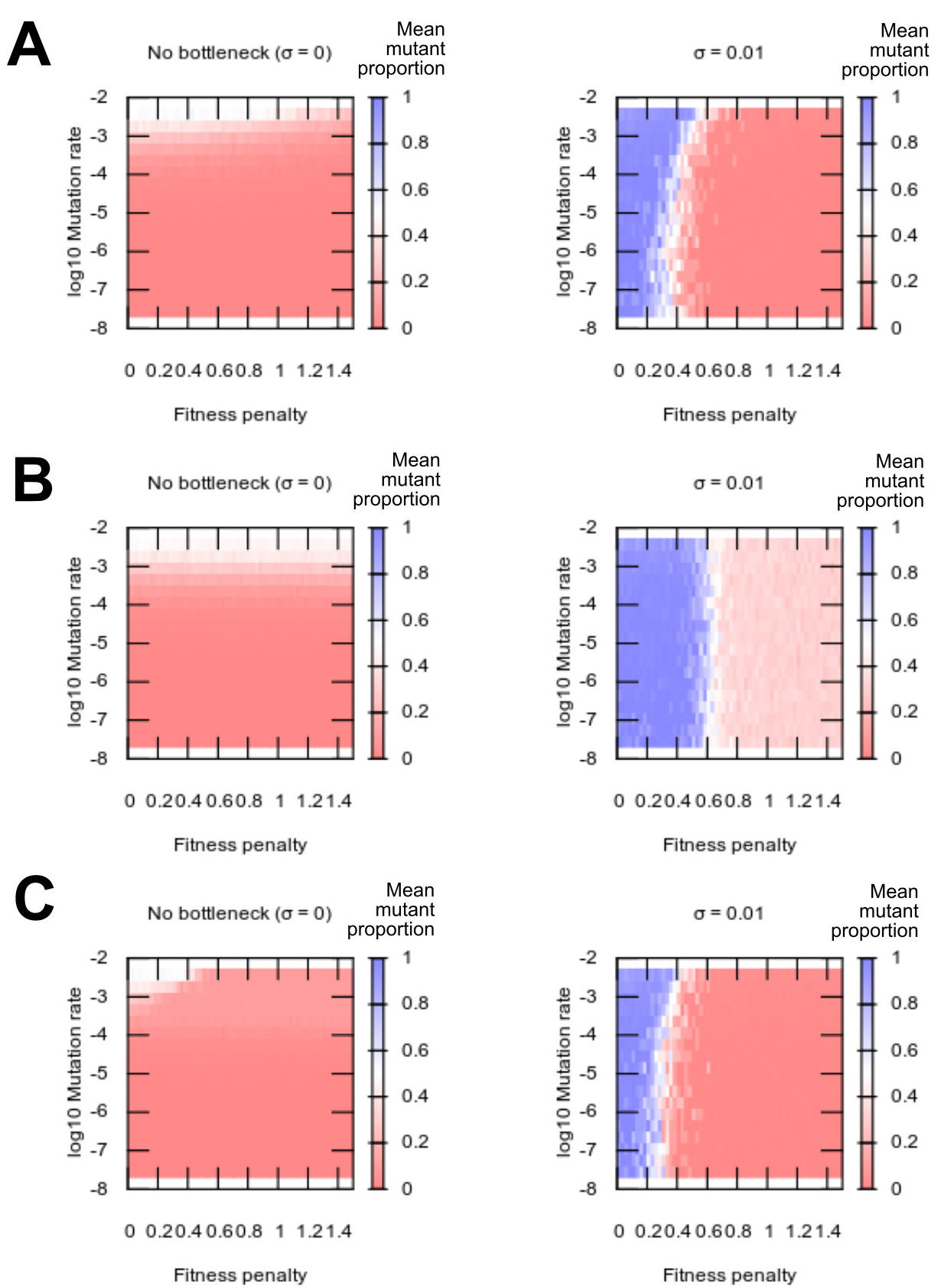

Supplement: Supplementary file 3 [file Image4.JPEG]

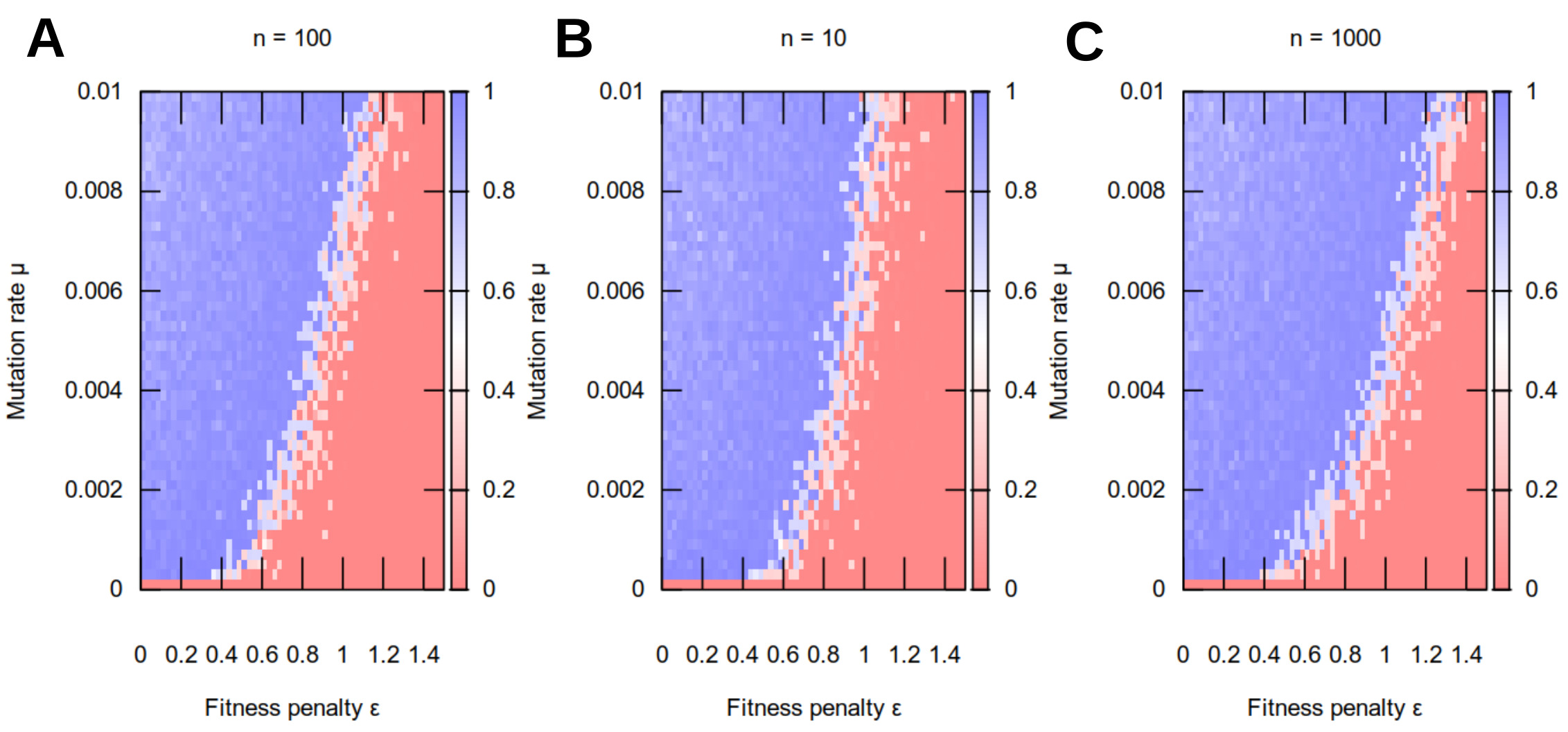

Supplement: Supplementary file 4 [file Image2.JPEG]

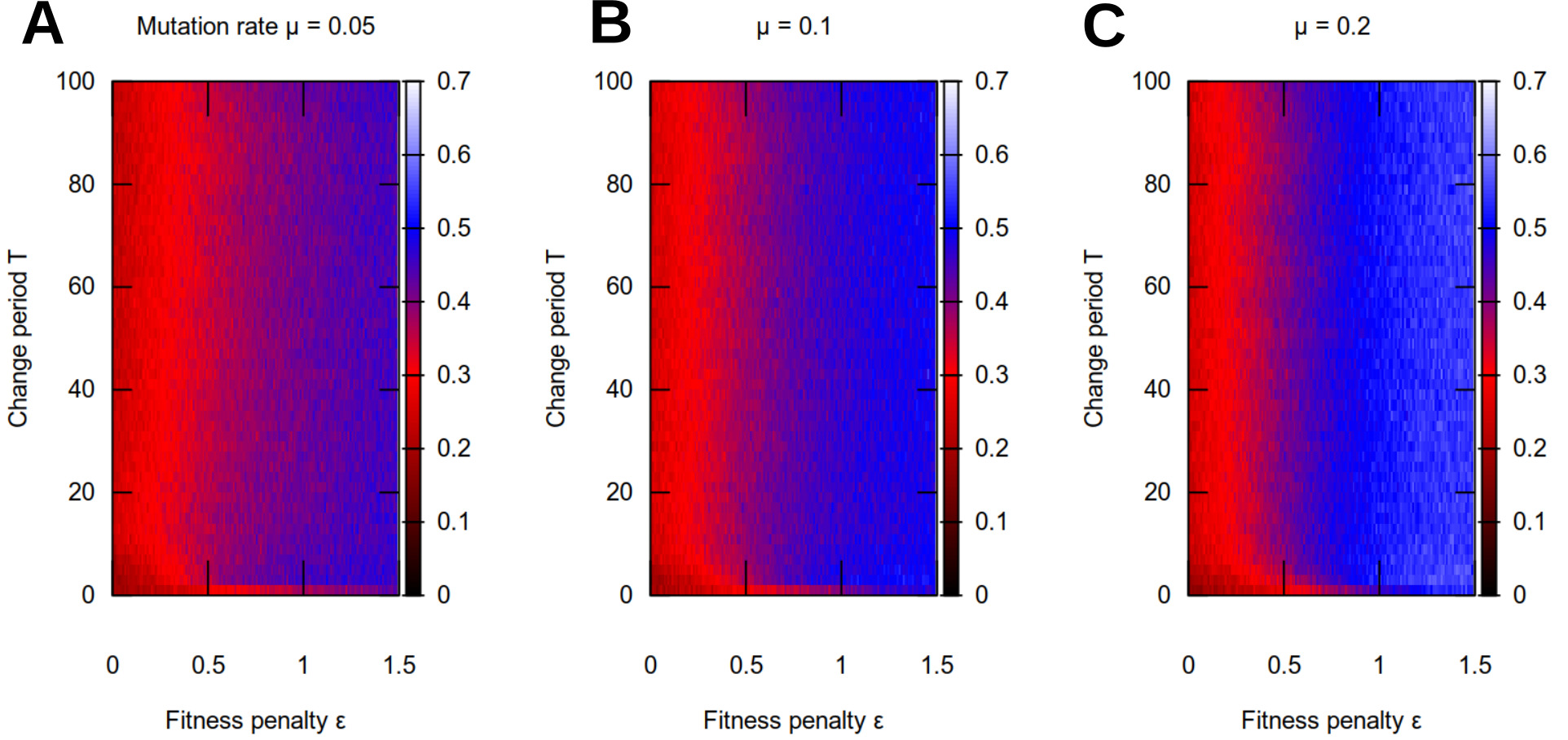

Supplement: Supplementary file 5 [file Image5.JPEG]
