# Supplementary material for: Organelle bottlenecks facilitate evolvability by traversing heteroplasmic fitness valleys
Source: Front Genet. 2022 Oct 28;13:974472. doi: 10.3389/fgene.2022.974472 (PMC9650085; doi:10.3389/fgene.2022.974472)
Supplement: Supplementary file 6 [file DataSheet1.pdf]

Supplementary Information

Derivation of approximate pertubation kernels for heteroplasmy statistics

We first consider two random variables W, M for the copy number of wildtype and mutant oDNAs in the cell. If a cell starts with copy numbers w, m respectively, the cumulative effects of mutations that occur during a generation is modelled by the following processes:

$$W = w - N_{WM}(w) + N_{MW}(m) \quad (1)$$
$$M = m + N_{WM}(w) - N_{MW}(m) \quad (2)$$

where $N_{ij}(.)$ are random variables describing the number of molecules of type i that are mutated to become molecules of type j. This model assumes a low mutation rate, so that double mutations are negligible. Given these definitions, we have for the expected values E, variances Var, and covariances Cov of the random variables involved:

$$E(W) = w - E(N_{WM}(w)) + E(N_{MW}(m)) \quad (3)$$
$$E(M) = m + E(N_{WM}(w)) - E(N_{MW}(m)) \quad (4)$$
$$Var(W) = Var(N_{WM}(w)) + Var(N_{MW}(m)) \quad (5)$$
$$Var(M) = Var(N_{WM}(w)) + Var(N_{MW}(m)) \quad (6)$$
$$Cov(W, M) = Cov(-N_{WM}(w), N_{WM}(w)) + Cov(N_{MW}(m), -N_{MW}(m)) \quad (7)$$
$$= -Var(N_{WM}(w)) - Var(N_{MW}(m)) \quad (8)$$
$$(9)$$

where the variance relations assume that N_{ij} are independent. We next assume that dynamics are Poissonian:

$$N_{WM}(w) \sim Po(\mu w) \quad (10)$$
$$N_{MW}(m) \sim Po(\mu m) \quad (11)$$
$$(12)$$

And, as $E(Po(\lambda)) = Var(Po(\lambda)) = \lambda$,

$$E(W) = w - \mu w + \mu m \quad (13)$$
$$E(M) = m + \mu w - \mu m \quad (14)$$
$$Var(W) = \mu w + \mu m \quad (15)$$
$$Var(M) = \mu w + \mu m \quad (16)$$
$$Cov(W, M) = -2\mu(w + m) \quad (17)$$
$$(18)$$

Let $H = M/(W + M)$ be heteroplasmy, and $h = m/(w + m)$ be the specific heteroplasmy value at the start of the

generation. Then, as $W + M = n$ remains constant,

$$
\begin{aligned}
E(H) &= E(M)/n && (19)\\
&= (hn + \mu(1-h)n - \mu hn)/n && (20)\\
&= h + \mu(1-2h) && (21)\\
Var(H) &= Var(M)/n^2 && (22)\\
&= (\mu(1-h)n + \mu hn)/n^2 && (23)\\
&= \mu/n && (24)
\end{aligned}
$$

and we use these moments in our approximate Normal mutation kernel. It will be seen that all these dynamics are neutral, with W and M behaving symmetrically – it is only at the population scale that selective differences, manifest through fitness differences, enter the model.

Incorporating dysfunctional oDNA types

For generality, we now consider a third type of oDNA, which is regarded as totally dysfunctional (contributing nothing to fitness). We now consider three random variables W, M, D for the copy number of wildtype, mutant, and dysfunctional oDNAs in the cell. If a cell starts with copy numbers w, m, d respectively, the cumulative effects of mutations that occur during a generation is now modelled by the following processes:

$$
\begin{aligned}
W &= w - N_{WM}(w) + N_{MW}(m) - N_{WD}(w) && (25)\\
M &= m + N_{WM}(w) - N_{MW}(m) - N_{MD}(m) && (26)\\
D &= d + N_{WD}(w) + N_{MD}(m). && (27)
\end{aligned}
$$

From these, as above,

$$
\begin{aligned}
E(W) &= w - E(N_{WM}(w)) + E(N_{MW}(m)) - E(N_{WD}(w)) && (28)\\
E(M) &= m + E(N_{WM}(w)) - E(N_{MW}(m)) - E(N_{MD}(m)) && (29)\\
E(D) &= d + E(N_{WD}(w)) + E(N_{MD}(m)) && (30)\\
Var(W) &= Var(N_{WM}(w)) + Var(N_{MW}(m)) + Var(N_{WD}(w)) && (31)\\
Var(M) &= Var(N_{WM}(w)) + Var(N_{MW}(m)) + Var(N_{MD}(m)) && (32)\\
Var(D) &= Var(N_{WD}(w)) + Var(N_{MD}(m)) && (33)\\
Cov(W, M) &= Cov(-N_{WM}(w), N_{WM}(w)) + Cov(N_{MW}(m), -N_{MW}(m)) && (34)\\
&= -Var(N_{WM}(w)) - Var(N_{MW}(m)) && (35)\\
Cov(W, D) &= Cov(-N_{WD}(w), N_{WD}(w)) && (36)\\
&= -Var(N_{WD}(w)) && (37)\\
Cov(M, D) &= Cov(-N_{MD}(m), N_{MD}(m)) && (38)\\
&= -Var(N_{MD}(m)) && (39)
\end{aligned}
$$

and Poisson dynamics gives

$$N_{WM}(w) \sim Po(\mu w) \tag{40}$$
$$N_{MW}(m) \sim Po(\mu m) \tag{41}$$
$$N_{WD}(w) \sim Po(\mu_D w) \tag{42}$$
$$N_{MD}(m) \sim Po(\mu_D m) \tag{43}$$

so

$$E(W) = w - \mu w + \mu m - \mu_D w \tag{44}$$
$$E(M) = m + \mu w - \mu m - \mu_D m \tag{45}$$
$$E(D) = d + \mu_D w + \mu_D m \tag{46}$$
$$Var(W) = \mu w + \mu m + \mu_D w \tag{47}$$
$$Var(M) = \mu w + \mu m + \mu_D m \tag{48}$$
$$Var(D) = \mu_D w + \mu_D m \tag{49}$$
$$Cov(W, M) = -2\mu(w + m) \tag{50}$$
$$Cov(W, D) = -\mu w \tag{51}$$
$$Cov(M, D) = -\mu m \tag{52}$$

Now, nonzero d and μ_D mean that $W + M$ can in general depart from $n = w + m$, as functional oDNAs become dysfunctional. Now we define $N_D = W + M + D$ and $H_D = D/N_D$, with $n_D = w + m + d$ and $h_D = d/(w + m + d)$ the specific values at the start of the generation. Then $w = (1 - h)(1 - h_D)n_D$, $m = h(1 - h_D)n_D$, $d = h_D n_D$. We approximate moments of H, now being the ratio of random variables, via Taylor expansions [**?**]. This approach is not without issues: as a ratio of random variables, H is a challenging quantity to treat, and in some circumstances the Taylor expansion picture (even beyond first order) does not fully capture its behaviour [**?**, **?**]. However, we employ it here to demonstrate the coarse-grained behaviour of the system. This approach gives us:

$$E(H) \simeq \frac{E(M)}{E(W) + E(M)} \tag{53}$$
$$= \frac{h(1 - h_D)n_D + \mu(1 - h)(1 - h_D)n_D - \mu h(1 - h_D)n_D - \mu_D h(1 - h_D)n_D}{(1 - \mu_D)(1 - h_D)n_D} \tag{54}$$
$$= \frac{h(2\mu + \mu_D - 1) - \mu}{\mu_D - 1} \tag{55}$$
$$Var(H) \simeq \frac{E(M)^2 Var(W) + E(W)^2 Var(M)}{(E(M) + E(W))^4} \tag{56}$$
$$= \frac{2(1 - 2h)^2\mu^3 + (1 + 2(h - 1)h)\mu(\mu_D - 1)^2) - h(h - 1)(\mu_D - 1)^2\mu_D + (1 - 2h)^2\mu^2(3\mu_D - 2)}{(h_D - 1)(\mu_D - 1)^4 n_D} \tag{57}$$
$$E(H_D) = E(D)/n_D \tag{58}$$
$$= h_D + \mu_D(1 - h_D) \tag{59}$$
$$Var(H_D) = Var(D)/n_D^2 \tag{60}$$
$$= \mu_D(1 - h_D)/n_D, \tag{61}$$

and use these moments in the normal perturbation kernels modelling the action of intergenerational mutations.
